# Induction of AHR Signaling in Response to the Indolimine Class of Microbial Stress Metabolites

**DOI:** 10.3390/metabo13090985

**Published:** 2023-08-31

**Authors:** Dhwani Patel, Iain A. Murray, Fangcong Dong, Andrew J. Annalora, Krishne Gowda, Denise M. Coslo, Jacek Krzeminski, Imhoi Koo, Fuhua Hao, Shantu G. Amin, Craig B. Marcus, Andrew D. Patterson, Gary H. Perdew

**Affiliations:** 1 Department of Biochemistry and Molecular Biology, The Pennsylvania State University, University Park, PA 16802, USA; 2 Department of Veterinary and Biomedical Sciences, Center for Molecular Toxicology and Carcinogenesis, The Pennsylvania State University, University Park, PA 16802, USA; 3 Department of Environmental and Molecular Toxicology, Oregon State University, Corvallis, OR 97331, USA; 4 Department of Pharmacology, Penn State College of Medicine, Hershey, PA 17033, USA

**Keywords:** Ah receptor, indolimine, CYP1A1, cancer, tryptophan, AHR

## Abstract

The aryl hydrocarbon receptor (AHR) is a ligand-activated transcription factor that plays an important role in gastrointestinal barrier function, tumorigenesis, and is an emerging drug target. The resident microbiota is capable of metabolizing tryptophan to metabolites that are AHR ligands (e.g., indole-3-acetate). Recently, a novel set of mutagenic tryptophan metabolites named indolimines have been identified that are produced by *M. morganii* in the gastrointestinal tract. Here, we determined that indolimine-200, -214, and -248 are direct AHR ligands that can induce *Cyp1a1* transcription and subsequent CYP1A1 enzymatic activity capable of metabolizing the carcinogen benzo(a)pyrene in microsomal assays. In addition, indolimines enhance *IL6* expression in a colonic tumor cell line in combination with cytokine treatment. The concentration of indolimine-248 that induces AHR transcriptional activity failed to increase DNA damage. These observations reveal an additional aspect of how indolimines may alter colonic tumorigenesis beyond mutagenic activity.

## 1. Introduction

The commensal microbiota and host invoke multiple mutualistic mechanisms to establish homeostasis, such as education of the immune system, enhanced digestion, and protection from pathogenic organisms. Indeed, communications between the host and the microbiota can occur through the secretion of chemical metabolites that are capable of readily diffusing either passively or actively across the mucosal and epithelial cell barrier in the intestinal tract. Often the bacterial species that produce a given metabolite and factors that regulate metabolite production are poorly understood. Gut bacterial metabolism of tryptophan generates a variety of metabolites, including indole, indole-3-acetate, and indole-3-propionate (IPA). The latter metabolite is produced by *Clostridium sporogenes* in the gut, is found in host serum at significant levels, and is a potent antioxidant that may have protective effects against human chronic diseases [1,2,3]. The physiological significance of other tryptophan metabolites produced by microorganisms is now only starting to emerge.

The intestinal epithelium expresses membrane and cytosolic receptors capable of sensing low-molecular-weight molecules made by the microbiota. An example is the bacterial production of short-chain fatty acids, which are cognate ligands for the free fatty acid receptors 2 and 3 (FFAR2/3) [4]. These receptors belong to the superfamily of G-protein-coupled receptors. FFAR2/3 are expressed in a variety of tissues and have been demonstrated to contribute to a variety of physiological and disease processes. Other classes of environmental sensors are xenobiotic receptors, including the aryl hydrocarbon receptor (AHR), pregnane X receptor (PXR), and constitutive androstane receptor (CAR), which are expressed in the intestinal tract. These receptors have distinct yet somewhat promiscuous ligand specificity, and the identity of bacterially produced small molecules that bind to these receptors continues to emerge [5].

The AHR is a ligand-activated bHLH-PAS transcription factor known to be a sensor of environmental cues, such as small molecules derived from gut-microorganism- or diet-derived small molecules. The AHR was first identified as the receptor that mediates the effects of 2,3,7,8-tetrachlorodibenzo-*p*-dioxin (TCDD). The hallmark of AHR agonist activity is the ability to heterodimerize with ARNT and bind to dioxin-responsive elements, resulting in enhanced CYP1A1 transcription [6]. Most initial studies focused on the role of the AHR in xenobiotic metabolism (e.g., polycyclic aromatic hydrocarbons) and TCDD-induced toxicity. With the availability of AHR knockout mice, the physiological role of the AHR was identified in a variety of activities, such as the modulation of immune cell phenotypes and epithelial cell differentiation both in the skin and the intestinal tract [7,8]. These observations indicate that the AHR is critical in the maintenance of barrier tissues and can respond to various chemical insults.

Diet, host, and microorganism-mediated tryptophan metabolism are potentially major sources of AHR ligands dependent on the tissue and local ligand production [9]. The gut microbiota are capable of metabolizing tryptophan into a series of metabolites. Some of these metabolites are AHR ligands, including indole, indole-3-acetic acid, 2-oxindole, tryptamine, and 2,8-dihydroxyquinoline. All are of bacterial origin, as determined by a comparison of metabolites in conventional versus germ-free mouse cecal contents [10]. Recent studies examining human serum revealed six relatively abundant tryptophan metabolites: indole-3-acetic acid, indole-3-lactic acid, indole-3-carboxaldehyde, kynurenic acid, kynurenine, and IPA [9]. Surprisingly, an examination of conventional mouse serum versus germ-free mouse serum revealed that the concentration of each compound was similar, except for IPA, which requires the presence of a microbiota. Thus, while the microbiota can make a variety of tryptophan metabolites, they appear to contribute little to systemic levels. Thus, when considering the context-specific contributions of indolimines to AHR activation in vivo, this would only occur in the presence of the other tryptophan metabolites. 

The screening of a collection of commensal bacteria identified several microbes that produce genotoxic small molecule metabolites, including *Morganella morganii*, found to be enriched in the gut microbiota of colorectal cancer patients. Large-scale cultivation of *M. morganii* and the isolation of specific genotoxic metabolites led to the identification of several indole derivatives, termed indolimines [11]. These metabolites appear to be produced through the synthesis of indole-3-aldehyde and condensation with a primary amine (e.g., phenethylamine) that requires the expression of an amino acid decarboxylase. These metabolites are structurally similar to other microbiota-derived tryptophan metabolites, many of which are AHR ligands [12]. Therefore, we tested whether indolimines are AHR ligands and found that all three indolimines are AHR agonists in human colonic tumor cells, capable of activating the AHR at nanomolar levels. These observations add an additional aspect to how these compounds may modulate colon carcinogenesis.

## 2. Material and Methods

### 2.1. Materials

Indolimine 200, 214, and 248 (I-200, I-214, and I-248) were synthesized, as described in supplementary methods; conformation of structural identity and purity were confirmed using NMR and LC-MS analysis (see Supplementary Figures S1–S4). The AHR photoaffinity ligand 2-azido-3-[^125^I]iodo-7,8-dibromodibenzo-*p*-dioxin ([^125^I]-PAL) was synthesized, as previously described [13]. Indirubin was purchased from Enzo Life Sciences. Benzo(*a*) pyrene (B(*a*)P), GNF351, and estradiol were obtained from Sigma. 9-hydroxy-benzo(a)pyrene was synthesized, as previously described [14].

### 2.2. Cell Culture

The HepG2 40/6 stable DRE-driven reporter line was generated and cultured, as previously described [15]. The Hepa 1.1 stable DRE-driven reporter cell line was obtained from Dr. Michael Denison (University of California, Davis, CA, USA) and cultured under the same conditions as HepG2 40/6 cells. The human epithelial colorectal adenocarcinoma cell line Caco-2 was obtained from ATCC and maintained in α-minimal essential medium (Sigma, Hong Kong, China; M0894), supplemented with 20% fetal bovine serum (Gemini), 100 U/mL penicillin, and 100 μg/mL streptomycin; for actual treatments, the medium was replaced by a medium containing 5% serum. HN30 cells were obtained and cultured, as previously described [16]. LS174T cells (ATCC #3521130) were cultured with α-minimal essential medium supplemented with 8% (*v*/*v*) fetal bovine serum, 100 U/mL penicillin, and 100 µg/mL streptomycin. All cells were grown in a humidified incubator at 37 °C, with 95% air and 5% CO_2_.

### 2.3. Capillary Immunoblotting Analysis

Cell cytosolic lysates and histone extracts were diluted to 0.5 mg/mL protein in 0.1X Sample Buffer (ProteinSimple, San Jose, CA, USA) and subjected to WES™ capillary system analysis. For cytosolic lysates, the primary antibody for CYP1A1 (Proteintech, 13241, 1:100) was visualized with the WES anti-rabbit secondary HRP-conjugated antibody (ProteinSimple) and normalized to total protein using the Total Protein Detection Module (ProteinSimple, DM-TP01). For quantification, the chemiluminescence peak area of CYP1A1 was normalized to the total protein peak area for each sample. To quantify the level of phosphorylated histone H2AX, the primary antibody for phospho-histone H2A.X (EMD Millipore #05-636; 1:100) was visualized with the WES anti-mouse secondary HRP conjugated antibody (ProteinSimple) and normalized to the peak area of histone H3 (Cell Signaling #4620; 1:100) visualized with WES anti-rabbit HRP Conjugate. 

### 2.4. RNA Isolation and mRNA Quantitation 

Isolation of RNA from Caco2 and LS174T cells and quantitation of specific mRNAs was performed, as previously described [17]. Briefly, 1 mL Tri Reagent (Sigma) was added to PBS-washed cells, and homogenates were transferred to 1.5 mL tubes. 

A volume of 0.2 mL chloroform (Sigma) was added to the homogenates, shaken vigorously for 15 s, and centrifuged (12,000× *g*, 15 min, 4 °C). Aqueous supernatants (0.4 mL) were transferred to fresh tubes and precipitated by addition of 0.5 mL 100% isopropanol (Fisher Chemical) followed by centrifugation (16,000× *g*, 30 min, 4 °C). Total RNA pellets were washed with 0.5 mL 70% ethanol (Fisher Chemical) and centrifuged (8000× *g*, 5 min, 4 °C). Total RNA pellets were air-dried and solubilized in 0.1 mL Ambion^TM^ DEPC-treated water (Invitrogen, Waltham, MA, USA). Total RNA concentration was determined using Nanodrop (Thermo Fisher Scientific, Waltham, MA, USA). Furthermore, 1 mg total RNA was converted to cDNA using a High-Capacity cDNA Reverse Transcription Kit, following the manufacturer’s instructions (Applied Biosystems, Waltham, MA, USA). Quantitative PCR was performed using PerFecTa SYBR green reagent (Thermo Fisher Scientific), following manufacturer’s instructions, on a CFX96 platform (BioRad, Hercules Scientific). Oligonucleotide primers (IDT DNA) utilized for quantitative PCR are presented in Supplementary Table S1.

### 2.5. Cell-Based AHR Ligand Competition Binding Assay

HN30 cells were utilized in this assay because of high levels of AHR expression. Cells were seeded into 12-well plates at 50,000 cells/well and cultured for 48 h in DMEM/F12 medium (Sigma) with 10% fetal bovine serum (Gemini BioProducts, West Sacramento, CA, USA), 25 mM HEPES, pH 7.4, and 100 U/mL penicillin and 100 μg/mL streptomycin. The medium was removed, and cells were washed with Dulbecco’s phosphate-buffered saline (PBS, Sigma). Cells in each well were incubated in 500 μL Hanks balanced salt solution (Gibco), 25 mM HEPES (pH 7.4), and 5 mg/mL bovine serum albumin for 30 min in an incubator. Next, cells were treated with AHR ligands, followed by the addition of 2 pmoles of [^125^I]-PAL, and cells were placed back in the incubator for 30 min. The medium was removed, 500 μL of PBS was added, and cells were exposed to UV light at >302 nm, at a distance of 8 cm, for 4 min using two 15-W UV lamps (Dazor Mfg. Corp. St. Louis, MO, USA). The PBS was removed, and cells lysed in 100 μL of 25 mM MOPS, 2 mM EDTA, 0.02% sodium azide, 10% glycerol, and 1% NP40. Lysates were transferred to microfuge tubes and centrifuged at 18,000× *g* for 20 min. Supernatants were subjected to tricine SDS-PAGE, and subsequently transferred to PVDF membrane. The radioactive AHR band was visualized with X-ray film, excised, and counted in a gamma counter.

### 2.6. CYP1A1/1B1 Microsomal Assay

Confluent Caco2 cells were treated with 5 nM TCDD for 24 h. Microsomes were isolated from cells washed with phosphate-buffered saline, trypsinized, and pelleted using centrifugation at 100× *g* for 3 min. The cell pellet was then washed with phosphate-buffered saline, pelleted, and resuspended in 0.25 M sucrose, 10 mM Tris-HCl (pH 7.5), with protease inhibitors. The cells were manually homogenized in a Dura-Grind Dounce-Type homogenizer (Wheaton, IL, USA). The cell homogenate was centrifuged at 10,000× *g* for 10 min at 4 °C, the supernatant was transferred and centrifuged at 100,000× *g* for 90 min at 4 °C. The resulting microsomal pellet was resuspended in the homogenization buffer, and the protein concentration was determined using BCA reagent kit (Pierce). Microsomes were stored at −80 °C for CYP1A1 activity assay. The P450-Glo CYP1A1/1B1 assay kit and NADPH regeneration system and luciferase assay kit were purchased from Promega (Promega, Madison, WI, USA) and utilized under the following conditions: 12.5 μL of CYP reaction mixture including microsomes, Luciferin-CEE and potassium phosphate buffer, and 12.5 μL of test compound in distilled water were combined and pre-incubated at 37 °C for 10 min in white 96-well plate. NADPH is required as a cofactor, and thus, reactions were initiated by adding an equal volume of NADPH regeneration system (25 μL) and placed at 37 °C for 25 min. In a final volume of 50 µL, reactions were performed in triplicate and contained 10 μg of microsomal protein in 100 mM K_2_PO_4_ (pH 7.4) buffer, 30 μM Luciferin-CEE, test compound, and NADPH regeneration system. Reactions were stopped by the addition of 50 μL of luciferin detection reagent and incubated at 37 °C for 10 min. Luminescence was measured using a luminometer, and the control without microsomes or with vehicle was used to assess background activity [18]. 

### 2.7. CYP1A1/1B1 Assays Coupled to MS Analysis

Microsomal assays were performed, as described in the previous section, except microsomes were isolated from Caco2 cells treated for 24 h with 20 μM I-248. The microsomal reactions were carried out at 37 °C in an incubator in the presence of 10 μM B(*a*)P and NADPH regeneration system and terminated by the addition of 800 µL ice-cold 100% methanol (*v*/*v*) containing 1 µM chlorpropamide as internal standard. These mixtures were kept at −20 °C for 30 min followed by centrifugation at 12,000× *g* for 20 min at 4 °C. The supernatants were dried under vacuum and reconstituted in 60 μL 50% methanol (*v*/*v*). After centrifugation, supernatants were transferred to autosampler vials for LC-MS/MS analysis, as previously described [19].

### 2.8. Computational Docking Analysis

The ligand-binding affinity of 3 indolimine analogs, including I-200, I-214, and I-248, was investigated using Autodock Tools 4 and Autodock Vina, and a cryo-EM structure-based computational model of the human AHR PAS B domain [9,20,21,22,23]. Docking models for the 3 indolimine ligands were built and minimized using PyMOL Molecular Graphics System, Version 2.52 Schrödinger, and LLC and all models were optimized for docking using Autodock Tools [24]. Autodock Vina was run using standard settings, using Grid box parameters generated in Autogrid for a 30 Å^3^ docking grid centered on the indirubin binding site, as described previously [25]. Docking results were analyzed using Autodock Tools 4 and The PyMOL Molecular Graphics System, Version 2.52. As shown in Supplementary Table S2, AHR docking model quality was validated using an Autodock Vina re-docking analysis with the structural coordinates of indirubin. RMSD re-docking error was calculated using the program LigRMSD, and the AHR PAS B domain cavity size was calculated using Caver Web 1.0 [26,27].

### 2.9. Histone Extraction and Protein Detection

LS174T cells (ATCC #3521130) were seeded in 6-well plates and treated for 24 h with 20 µM I-248 or 10 µM B(a)P in maintenance media. The cells were harvested following the histone extraction protocol for Western blot analysis (Abcam, Boston, MA, USA) with modifications. Briefly, the media were collected and centrifuged at 650× *g* for 10 min at 4 °C to pellet cells, and the supernatant was discarded. The cell pellet was washed twice with ice-cold PBS and then stored on ice. The attached cells were washed twice with ice-cold PBS. Then, 300 µL of Triton Extraction Buffer (TEB: PBS containing 0.5% Triton X 100 (*v*/*v*), 0.02% sodium azide, pH 7.4, adding protease and phosphatase inhibitors immediately before use) was added directly to each well. Cells were scraped and transferred to the corresponding tube containing the cell pellets from the media. To lyse the cells, they were incubated on ice for 10 min with mixing every 3 min. Samples were centrifuged at 650× *g* for 10 min at 4 °C to spin down the nuclei. The supernatant containing cytosolic protein was transferred to a new tube and stored for protein analysis. The nuclei pellets were washed once with 150 µL of TEB buffer and centrifuged. The supernatant was discarded, and the pellet was resuspended in 30 µL of 0.2 N hydrochloric acid. After overnight acid extraction of the histones at 4 °C, the samples were centrifuged at 650× *g* for 10 min at 4 °C to pellet debris. The supernatant containing the histones was transferred to a fresh tube and neutralized with 3 µL of 2N sodium hydroxide. Protein concentrations for both the cytosolic lysate and the histone extracts were determined using the BCA Protein Assay Kit (Pierce). Samples were stored at −80 °C. 

### 2.10. Viability Assay

LS174T cells were seeded in a 96-well plate and treated with 20 µM I-248 or 10 µM B(*a*)P solubilized in DMSO for 24 h. A Cell Counting Kit-8 (CCK-8: Sigma-Aldrich) was utilized to determine viability following the manufacture’s protocol. Briefly, 10 µL of CCK-8 solution was added to each well and incubated for 4 h. The absorbance at 450 nm was measured using a microplate reader. Percentage viability was compared to the vehicle (VEH) control. 

### 2.11. Data Analysis

Statistical analysis was performed using one-way analysis of variance with Tukey’s multiple comparisons post-test using Prism (version 9) software (GraphPad Software, Inc., San Diego, CA, USA). Data points and error bars represent the mean ± SEM of three independent biological determinations. Significance is expressed either * (*p* < 0.05), ** (*p* < 0.01), or *** (*p* < 0.001). Alphabetical characters signify statistical comparisons between two groups. All experiments were performed at least two times. 

## 3. Results

### 3.1. Indolimines Activate the AHR 

**Figure 1:**
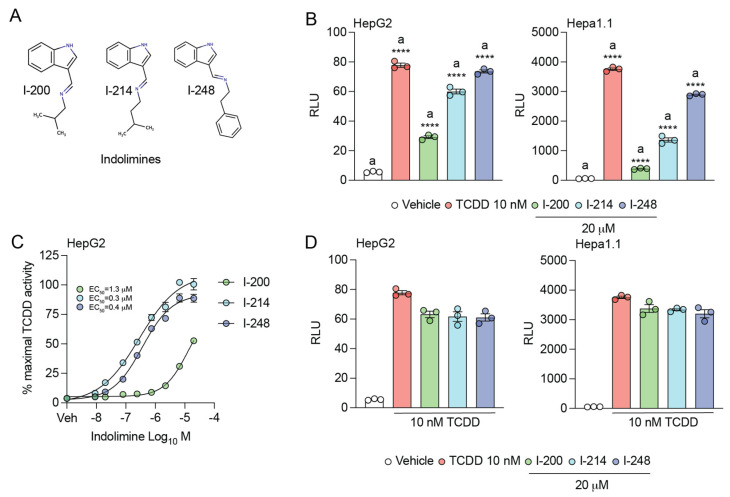
Genotoxic indolimines dose-dependently induce AHR transcriptional activity. (**A**) Structures of indolimines I-200, I-214, and I-248. (**B**) Human HepG2 40/6 or mouse Hepa 1.1 hepatoma cell lines harboring AHR-sensitive luciferase reporters were treated for 5 h with vehicle (DMSO), 10 nM TCDD, or 20 μM indolimine (I-200, I-214, or I-248). Cells were lysed, and AHR-dependent luciferase activity was quantified. Data represent mean relative luciferase units (RLUs) ± SEM (*n =* 3/group). Statistical significance was determined using one-way ANOVA coupled with Dunnett’s multiple comparisons test. (**C**) HepG2 cells were treated for 5 h with vehicle, 10 nM TCDD, or the indicated concentrations of I-200, I-214, or I-248. Cells were lysed, and AHR-dependent luciferase activity was quantified. Data represent mean percentage of AHR activity ± SEM (*n =* 3/group) relative to TCDD-induced response. (**D**) HepG2 and Hepa1.1 cells were treated for 5 h with vehicle, 10 nM TCDD, or 20 μM indolimine in combination with TCDD. Cells were lysed, and AHR-dependent luciferase activity was quantified. Data represent mean relative luciferase units (RLUs) ± SEM (*n =* 3/group). Statistical significance was determined using one-way ANOVA coupled with Dunnett’s multiple comparisons test. **** *p* < 0.0001.

Indolimines are produced by the bacterial metabolism of tryptophan and are structurally similar to other tryptophan metabolites found in the gut microbiome that are AHR agonists [9,28]. Therefore, we tested whether I-200, I-214, or I-248 (Figure 1A) are AHR agonists. All three indolimines exhibited significant agonist activity at 20 μM, with I-200 exhibiting the least activation potential in both a human HepG2 40/6 and mouse Hepa 1.1 dioxin-response-element-driven stable reporter cell lines (Figure 1B). Next, a dose–response experiment was performed to determine relative AHR agonist potency in the HepG2 40/6 reporter cell line (Figure 1C). I-248 and I-214 exhibited similar potency with an EC_50_ of ~0.30 μM, while the EC_50_ of I-200 was ~1.3 μM. Each indolimine failed to exhibit significant antagonism of TCDD agonist activity, consistent with the theory that indolimines are AHR agonists (Figure 1D).

### 3.2. Indolimines Induce AHR Target Gene Expression

**Figure 2:**
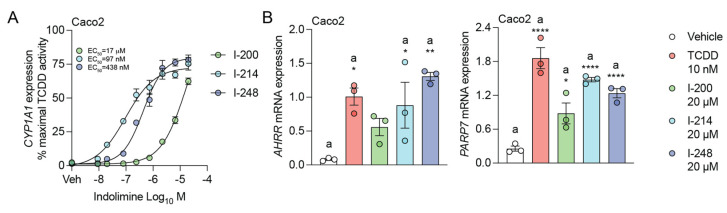
Indolimines dose-dependently induce transcription of AHR-responsive genes. (**A**) Human colonic Caco2 cells were treated for 4 h with vehicle, 10 nM TCDD, or the indicated concentrations of I-200, I-214, or I-248. Quantitative PCR analysis for *CYP1A1* expression was performed. (**B**) Caco2 cells were treated for 4 h with vehicle, 10 nM TCDD, or 20 μM indolimine (I-200, I-214, or I-248). Quantitative PCR analyses for *AHRR* and *PARP7* expression were performed. Quantitative PCR analysis for *IL6* was performed. Data represent mean relative expression (normalized to *ACTB*) ± SEM (*n =* 3/group). Statistical significance was determined using one-way ANOVA coupled with Dunnett’s multiple comparisons test. * *p* < 0.05, ** *p* < 0.01, **** *p* < 0.0001.

Caco2 cells were treated with increasing concentrations of indolimines, and the relative levels of *CYP1A1* mRNA were determined (Figure 2A). The EC_50_ for I-200, I-214, and I-248 *CYP1A1* mRNA levels was calculated to be 17 μM, 97 nM, and 438 nM, respectively. The indolimines induced increased expression of two additional direct AHR targets, *AHRR* and *PARP7*, in Caco2 cells, confirming that indolimines activate a spectrum of gene activation seen with other AHR ligands (Figure 2B). 

### 3.3. Indolimines Induce Combinatorial Activation of IL6 Expression

**Figure 3:**
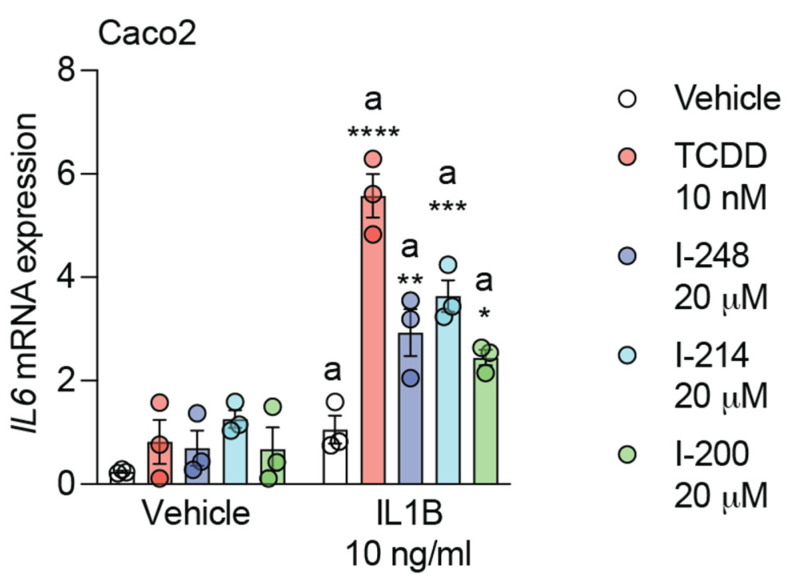
Indolimines in combination with IL1B enhance IL6 expression. Caco2 cells were treated for 4 h with vehicle, 10 nM TCDD, or 20 μM indolimine (I-200, I-214, or I-248) in isolation or in combination with 10 ng/mL human IL1B. Quantitative PCR analysis for *IL6* was performed. Data represent mean relative expression (normalized to *ACTB*) ± SEM (*n =* 3/group). Statistical significance was determined using one-way ANOVA coupled with Dunnett’s multiple comparisons test. * *p* < 0.05, ** *p* < 0.01, *** *p* < 0.001, **** *p* < 0.0001.

We have previously established that AHR activation enhances IL6 expression in combination with cytokine treatment [29]. The mechanism of this effect was determined to involve AHR occupation of dioxin response elements ~3 kB upstream of a proximal NFkB response element [30]. The presence of AHR/ARNT led to the displacement of HDAC1/3 and subsequent acetylation of p65. This would suggest a different mechanism of gene expression compared with *CYP1A1*. All three indolimines in combination with IL1B exhibit enhanced *IL6* expression compared with IL1B treatment alone in Caco2 cells (Figure 3). Thus, indolimines can contribute to the combinatorial regulation of IL6, similar to the effect observed with the potent AHR ligand TCDD.

### 3.4. Indolimines Are Direct AHR Ligands (Modeling Data)

**Figure 4:**
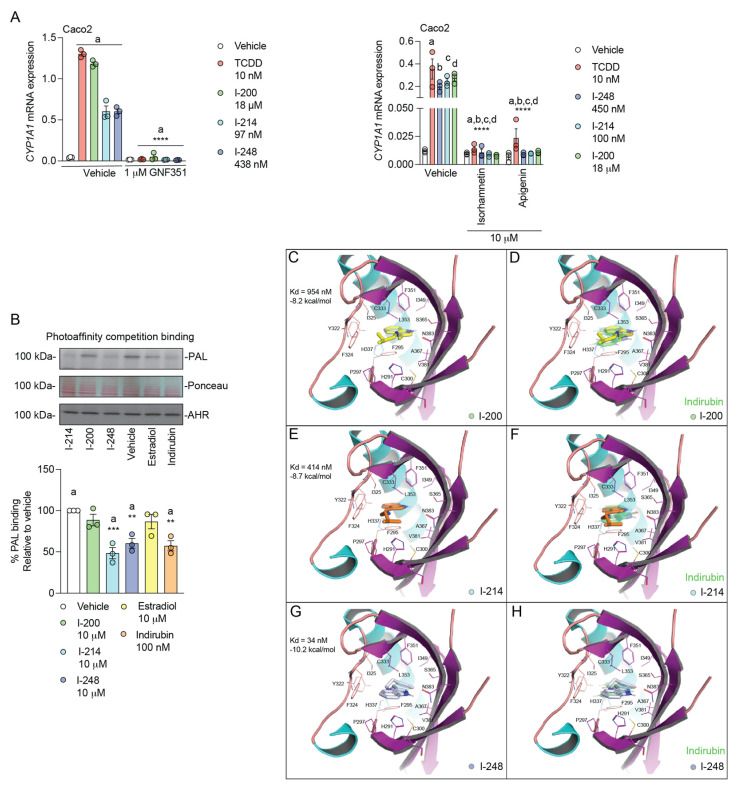
Indolimine-mediated AHR activity is a consequence of AHR binding. (**A**) Caco2 cells were treated for 4 h with vehicle, 10 nM TCDD or EC_50_ concentration of indolimines (18 µM I-200, 97 nM I-214, or 438 nM I-248) in isolation or in combination with 1 µM GNF351 AHR antagonist. In a separate experiment, Caco2 cells were also treated with vehicle, 10 nM TCDD, I-248, I-214, I-200 in the presence or absence of either isorhamnetin (10 µM) or apigenin (10 µM) for 24 h. Quantitative PCR analysis for *CYP1A1* was performed. Data represent mean relative expression (normalized to *ACTB*) ± SEM (*n =* 3/group). Statistical significance was determined using one-way ANOVA coupled with Dunnett’s multiple comparisons test. (**B**) Photoaffinity ligand competition assays were performed using human HN30 cells. Cells were pre-incubated with 10 µM indolimines (I-200, I-214, or I-248), 100 nM indirubin (positive control), or 10 µM estradiol (negative control) prior to addition of 4 nM AHR ^125^I-photoaffinity ligand (PAL). After 30 min, UV-mediated PAL cross-linking was performed, and cells were lysed. Lysates were resolved using SDS-PAGE. Specific PAL-labeled AHR bands were visualized through autoradiography and subsequently quantified by phosphor-imaging densitometry. The total protein content of lysates was visualized by Ponceau Red staining. Quantification of AHR protein was achieved through immunoblotting. Data represent mean percent PAL binding ± SEM relative to vehicle-treated controls (*n =* 3/group). Statistical significance was determined using one-way ANOVA coupled with Dunnett’s multiple comparisons test. (**C**–**H**) Computational docking analyses of indolimines ((**C**) I-200, (**E**) I-214, and (**G**) I-248) are presented in the closed form of the human PASB-AHR, derived from the cryo-EM structure of indirubin-bound HSP90-XAP2-AHR complex. (**D**,**F**,**H**) Comparison between I-200, I-214, I-248 and indirubin orientations within the PASB ligand-binding pocket. ** *p* < 0.01, *** *p* < 0.001, **** *p* < 0.0001.

While these data firmly established that the indolimines are AHR agonists, they do not demonstrate direct binding to the ligand-binding pocket of the AHR. If indolimines bind directly to the AHR to induce *CYP1A1* expression, then cotreatment with an AHR antagonist should block *CYP1A1* induction. Figure 4A reveals that GNF351 and two dietary flavonoids, which are all AHR antagonists, efficiently block CYP1A1 expression mediated by both 10 nM TCDD and the indolimines co-treated at the EC_50_ dose capable of inducing *CYP1A1*. Next, a cell-based AHR ligand-binding competition assay was performed, which employed a [^125^I]-PAL (Figure 4B). The HNSCC cell line HN30 was co-treated with indolimines or the potent AHR ligand indirubin and [^125^I]-PAL. After 1 h, the cells were exposed to UV light, inducing the covalent binding of the photoligand to the AHR. Western blot analysis demonstrated that there are similar levels of AHR and protein on the blot after each treatment. Cells co-treated with I-214, I-248 and indirubin exhibited significantly reduced levels of [^125^I]-PAL incorporation, suggesting direct competition for the ligand-binding pocket. In contrast, estradiol, which is not an AHR ligand, failed to reduce [^125^I]-PAL incorporation into the AHR compared to vehicle, consistent with the concept of the specific inhibition of [^125^I]-PAL binding mediated by indolimines. To provide additional evidence that indolimines bind to the ligand-binding pocket, we turned to computational docking analysis. All three indolimine analogs (200, 214, and 248) can bind to the PAS B domain in the nanomolar affinity range, in the same primary-ligand-binding pocket of the human AHR, as defined by the high-affinity AHR ligand indirubin in the cryo-EM structure [23]. As shown in Figure 4C, I-200, which has the shortest side chain of the three analogs tested, binds the AHR with high nanomolar affinity (K_D_ = 954 nM; −8.2 kcal/mol; See Table 1) at essentially the same active-site location as indirubin (Figure 4D). Indolimine-214, which has one additional carbon atom in its side chain compared to Indolimine-200, docks the PAS B domain with more than twofold greater affinity than Indolimine-200 (K_D_ = 414 nM; −8.7 kcal/mol; Figure 4E), within the same hydrophobic pocket as indirubin (Figure 4F). Indolimine-248, which has the largest side chain modification of the series tested, displayed the highest affinity toward the PAS B domain, docking in the low nanomolar affinity range (K_D_ = 34 nM; −10.2 kcal/mol; Figure 4G). In this configuration, I-248 binds the PAS B domain in essentially the same hydrophobic groove as the prototype ligand, indirubin, but the fit appears tight, as the ligand’s linker region must pucker slightly to allow the proper positioning of the heterocyclic rings (Figure 4H). Importantly, the relative affinity differences between the indolimines determined by modeling agree with the rank order of AHR activation potential observed in Figure 1B, further supporting that indolimines are direct AHR ligands.

### 3.5. Indolimines Increase CYP1A1 Protein Expression and Carcinogen Metabolism 

**Figure 5:**
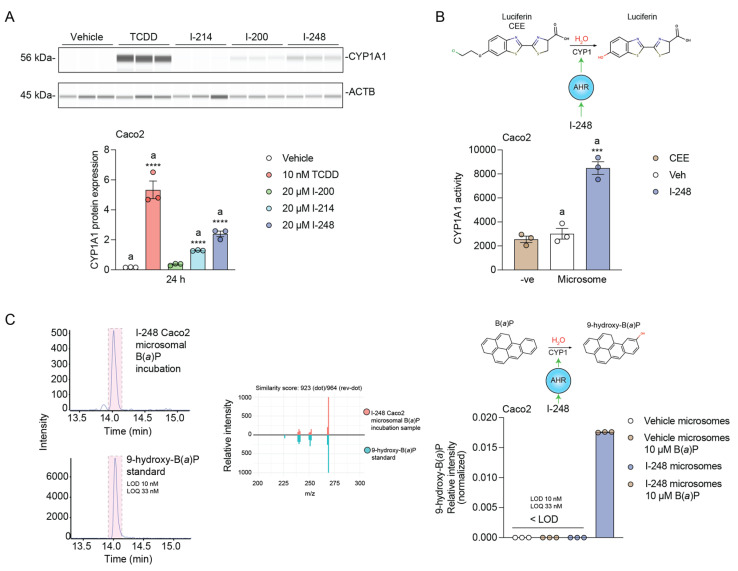
Indolimines promote AHR-dependent CYP1-mediated biotransformation. (**A**) Caco2 cells were treated for 24 h with vehicle, 10 nM TCDD, or 20 μM indolimine (I-200, I-214, or I-248). Quantitative CYP1A1 protein expression was performed. (**B**) Caco2 cells were treated with vehicle or 20 µM I-248 for 24 h to induce microsomal CYP1A1 protein expression. Vehicle and I-248-induced microsomes were isolated, and CYP1A1 enzymatic activity over 45 min was quantified using Luciferin-CEE as a CYP1A1-specific substrate in combination with NADPH regeneration system. Luciferin-CEE in the absence of microsomal protein was used as the negative control. (**C**) Vehicle and 20 µM I-248 24-h-induced Caco2 microsomal protein samples were incubated with 10 µM B(a)P and NADPH regenerating system for 2 h at 37 °C. Reaction products were extracted in 100% methanol and analyzed using LC/MS in parallel with known B(a)P metabolite standards. The presence and identity of 9-hydroxy-B(a)P in I-248-induced microsome reaction extracts were validated by chromatographic and mirror MS/MS comparison with 9-hydroxy-B(a)P standard. Relative abundance of 9-hydroxy-B(a)P in I-248-induced microsome extracts is indicated. 9-hydroxy-B(a)P in vehicle microsomes incubated with B(a)P and I-248-induced microsomes incubated without B(a)P were below the limit of detection (LOD), which was determined to be 10 nM. Data represent mean relative CYP1A1 activity ± SEM (*n =* 3/group). Statistical significance was determined using one-way ANOVA coupled with Dunnett’s multiple comparisons test. *** *p* < 0.001, **** *p* < 0.0001.

Caco2 cells were treated with each indolimine or TCDD for 24 h, and the level of microsomal CYP1A1 was assessed by WES analysis. The level of CYP1A1 expression induced by the indolimines revealed the highest level of expression with I-248 (Figure 5A), consistent with the relative level of CYP1A1 mRNA observed in Figure 2. TCDD was utilized as the positive control. Microsomes isolated from the control and I-248-treated Caco2 cells were tested for the ability to metabolize the CYP1A1-specific substrate luciferin-CEE. Microsomes from I-248 cells mediated significant metabolic activity (Figure 5B). In contrast, control microsomes exhibited essentially no increase in activity when compared to the substrate-only control. Next, microsomes from I-248 cells were incubated with the potent carcinogen B(a)P, and the presence of metabolites was assessed. Only one significant metabolite peak was observed, and the LC-MS/MS spectra matched a 9-hydroxy-B(a)P standard (Figure 5C). These results indicate that I-248 is capable of inducing carcinogen metabolism in a human colonic carcinoma line.

### 3.6. I-248 Fails to Induce DNA Damage at a Concentration That Induces CYP1A1 Expression

**Figure 6:**
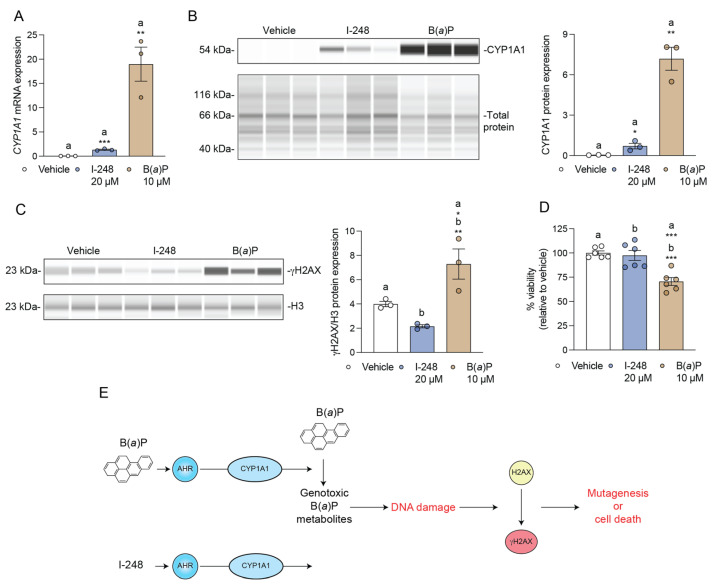
B(a)P, but not I-248, mediates DNA damage response. (**A**,**B**) LS174T cells were treated for 24 h with vehicle, I-248, or B(a)P, and the level of *CYP1A1* expression or CYP1A1 protein levels were assessed. Quantitative PCR analysis for *CYP1A1* expression was performed. Data represent mean relative *CYP1A1* expression (normalized to *ACTB*) ± SEM (*n =* 3/group). Statistical significance was determined using Student’s *t*-test. (**C**) Scheme depicting B(a)P-mediated DNA damage. B(a)P, but not I-248, increases DNA damage in LS174T cells, as assessed by the level of γH2AX formation after 24 h exposure. Data represent mean relative γH2AX (normalized to histone H3) ± SEM. (**D**) Viability of LS174T cells after 24 h exposure to indicated treatments. Statistical significance was determined using one-way ANOVA with Dunnett’s multiple comparison test. * *p* < 0.05, ** *p* < 0.01, *** *p* < 0.001. (**E**) Schematic summary of Figure 6.

**Figure 7:**
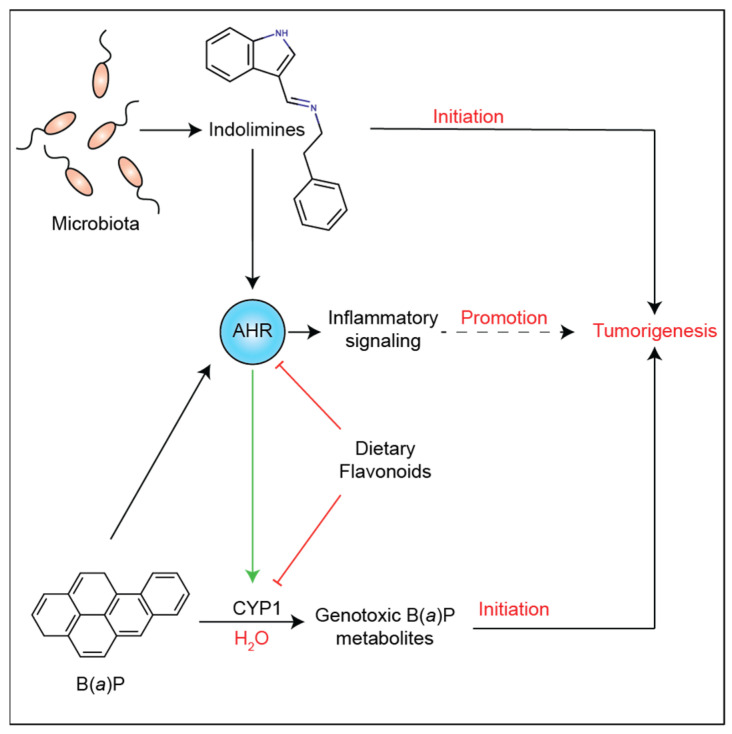
Overall scheme of the role of indolimines in colonic tumorigenesis. The AHR is proposed to mediate both carcinogen metabolism and inflammatory signaling within the tumor microenvironment.

LS174T is a p53 wild-type human colorectal cell line that is appropriate for examining DNA damage responses, while Caco2 cells do not express wild-type p53 [31]. *CYP1A1* mRNA and CYP1A1 protein are induced after 24 h treatment with I-248 or B(a)P, confirming that LS174T cells are AHR-responsive cells (Figure 6A,B). The ability of I-248 and B(a)P to induce DNA damage was assessed using γH2AX, a marker of DNA breaks. After 24 h, B(a)P induced a robust increase in DNA damage in LS174T cells (Figure 6C). In contrast, I-248 failed to increase the level of γH2AX at a concentration that leads to a marked increase in AHR activation. Under the same experimental conditions, a viability assay revealed that only B(a)P decreased LS174T cell viability (Figure 6D). The results are summarized in Figure 7.

## 4. Discussion

Indolimines are likely synthesized in the gut through microbial metabolism of a primary amine and indole-3-aldehyde [11]. Indole-3-aldehyde is a tryptophan metabolite and acts as a relatively weak AHR ligand with an EC_25_ of 52.4 μM in a Hep G2 40/6 AHR reporter cell line, compared to I-214 and I-248, with EC_25_ values of 0.05 and 0.10 μM, respectively [9]. Human AHR docking studies reveal the same trend in binding potential [9]. These findings suggest that indolimines are relatively potent ligands, compared to other abundant tryptophan microbial metabolites found in the gut, such as indole-3-aldehyde, indole-3-propionic acid, and indole-3-acetic acid. Interestingly, primary amines derived from amino acids are common in food products, particularly fermented foods [32,33]. Indole-3-aldehyde can be produced by bacteria during food fermentations [34]. This expands the potential sources of not only reported indolimines but other possible indolimines from an array of primary amines. In addition, the consumption of primary amines could lead to indolimine formation in the gut. Thus, there may be yet other novel AHR ligands that remain to be identified.

Numerous studies have demonstrated that AHR activation is an important mechanism for maintaining intestinal homeostasis, influencing multiple cell types within the gastrointestinal tract [35]. For example, the activation of the AHR increases the expression of IL-22 from group 3 innate lymphoid cells, which protects intestinal stem cells from genotoxic stress [36]. Furthermore, AHR activation coordinates intestinal stem cell lineage programming and can lead to an increase in goblet cells and the overall inhibition of epithelial cell proliferation [17]. Studies have also shown that AHR expression or activation attenuates colitis in mouse models [37,38]. The lack of global AHR expression enhances colon carcinogenesis in mice. In addition, the activation of the AHR can exhibit a chemo-preventive effect on colon carcinogenesis [39]. Both outcomes are consistent with the overall concept of AHR-mediated maintenance of intestinal homeostasis, which would likely inhibit the early stages of carcinogenesis. However, once a tumor is formed, what is less clear is the role of the AHR within the tumor microenvironment. A common feature of many tumors is a relatively high level of AHR expression compared to normal tissue [40,41]. Patients with high AHR and kynurenine levels are associated with reduced overall survival from glioblastoma, perhaps through an immunosuppression mechanism [42]. Thus, in the context of existing colonic tumors, the presence of *M. morganii*-producing indolimines could contribute to AHR activation in combination with other tryptophan metabolites, such as kynurenine, in the intestinal tumor microenvironment, likely leading to enhanced tumor outgrowth and immune suppression.

The AHR may contribute to inflammatory signaling within the tumor microenvironment through the combinatorial regulation of several chemokine and cytokine genes [29,43,44]. For example, the AHR upon activation and heterodimerization with ARNT has been shown to directly bind to DRE in the upstream regulatory region of the IL6 promoter and, in combination with cytokine signaling, can lead to the synergistic induction of IL6 [29,30]. Chromatin immunoprecipitation assays revealed that, upon TCDD and IL1B co-treatment, the AHR is recruited to ~3 kb from the transcriptional start site, and NFkB is recruited to the proximal promoter. Furthermore, the presence of the AHR on the promoter leads to the dismissal of HDAC1, which correlates to hyperacetylation of p65. This latter observation has been shown to lead to increased p50 transcriptional activity. This mode of gene regulation would be consistent with the concept that the AHR is an external sensor of the microbiota that plays an important role in the response to infection, particularly in barrier tissues. However, in a tumor microenvironment AHR activation could lead to enhanced inflammatory signaling, which would likely be pro-survival [45]. Furthermore, the combination of AHR and cytokine signaling has been demonstrated to enhance growth factor expression in carcinoma cell lines [16]. The ability of the AHR to enhance the expression of certain cytokines, chemokines, and growth factors and the results presented here, in Caco2 cells, support the hypothesis that indolimines may enhance tumorigenesis through a non-genotoxic mechanism.

Many bioactivated carcinogens share common structural features with potent AHR ligands, such as those that contain multiple aromatic rings with a highly reactive functional group and are planar hydrophobic structures. These same attributes are also common to CYP1A1/1B1 substrates. Polycyclic aromatic hydrocarbons (e.g., B(a)P) and heterocyclic amines (e.g., 2-amino-3-methyl-9H-pyrido [2,3-b]indole) are prime examples of carcinogens that are AHR agonists that induce CYP1A1 expression, which leads to their metabolism to more polar products [46,47]. While this scenario is considered a mechanism to clear hydrophobic compounds, it can also lead to the formation of reactive intermediates, which is the case with B(a)P. CYP1A1 metabolism of B(a)P can lead to the formation of the reactive benzo[a]pyrene-7,8-dihydrodiol-9,10-epoxide. Another genotoxic intermediate of B(a)P is benzo(a)pyrene-7,8-dione (BPQ), which is generated by aldo-keto reductase. BPQ is an AHR agonist and, thus, is capable of inducing CYP1A1 expression [48]. In addition, BPQ can be transported by the AHR into the nucleus due to the ability of the AHR to undergo nucleocytoplasmic shuttling, leading to increased DNA damage [49]. This would suggest that indolimines may use the same mechanism to translocate into the nucleus. However, the presence of an AHR antagonist in the diet, such as the flavonoid apigenin, could possibly block this mechanism of indolimine translocation into the nucleus, presenting another theory that would require further study. In addition to indolimine-mediated mutagenic activity, indolimine activation of the AHR would likely alter the metabolism of dietary carcinogens, such as polycyclic aromatic hydrocarbons. Overall, this study determined that indolimines made through the condensation of a primary amine and indole-3-aldehyde generate a previously unidentified class of AHR ligands. This study suggests that other primary amines may undergo condensation with various aldehydes, leading to a complex array of potential AHR ligands. Indolimines can be added to the growing list of endogenously available tryptophan metabolites capable of activating the AHR. Future studies will examine the physiological and pathologic consequences of indolimine-mediated AHR activation.

## Figures and Tables

**Table 1:** Computational Docking Analysis of Indolimines in the Human AHR PAS B Domain.

Substrate		Autodock Vina	
		*Structure-based Homology Model*	
		Human AHR PAS B domain	
		Binding Energy ^a^ (kcal/mole)	Dissociation Constant ^b^ *[K_D_] nM*
**Indolimine-200**	*Max* ^c^	**−8.2**	***953.9***
	*Avg* ^d^	*−7.5 ± 0.65*	*5515 ± 8391*
**Indolimine-214**	*Max*	**−8.7**	***413.5***
	*Avg*	*−8.4 ± 0.34*	*865 ± 572*
**Indolimine-248**	*Max*	**−10.2**	***33.7***
	*Avg*	*−9.9 ± 0.42*	*69.7 ± 57.5*

^a^ Substrate binding energies were derived computationally using Autodock Vina. ^b^ Dissociation binding constants (K_D_) (at a micromolar (µM) scale) were derived computationally from Autodock Vina data using the Autodock 4.2 inhibitory constant conversion scale, based on the equation: y = 0.5982ln(x) − 12.304. ^c^ Max refers to the low-energy Vina docking solution (in kcal/mole), with the highest predicted binding affinity (K_D_) for the AHR PAS B domain. If multiple results had the same binding energy, the pose with the closest contact distance to the heme center was selected. ^d^ Avg refers to the average or cumulative dissociation constant and binding energy (± the standard deviation) for the Vina docking poses obtained for each individual substrate:model docking combination (N < 10).

## Data Availability

Data are contained within the article or Supplementary Materials.

## Supplementary Materials

The following supporting information can be downloaded at: https://www.mdpi.com/article/10.3390/metabo13090985/s1, chemistry methods and indolimine synthesis; Figure S1: Identification of synthetic indolimines using LC-MS/MS; Figure S2: Conformation of the structure of indolimine-200 using NMR; Figure S3: Conformation of the structure of indolimine-214 using NMR. Figure S4: Conformation of the structure of indolimine-248 using NMR; Table S1: Primer sequences utilized in qRT-PCR analysis; Table S2: Computational validation of the human AHR PAS-B domain model. Scheme S1: Synthesis of indolimines.
