# Supplementary material for: Induction of AHR Signaling in Response to the Indolimine Class of Microbial Stress Metabolites
Source: Metabolites. 2023 Aug 31;13(9):985. doi: 10.3390/metabo13090985 (PMC10535990; doi:10.3390/metabo13090985)
Supplement: Supplementary file 1 [file metabolites-13-00985-s001.zip › metabolites-2570071-supplementary.pdf]

**Supplemental Methods**

**General chemistry methods**

**LC/MS method section.** Reagents and solvents were purchased from commercial vendors and were of the highest purity available and used without further purification unless otherwise noted. Qualitative/quantitative analysis was performed by reverse phase UHPLC using a Prominence 20 UFLCXR system (Shimadzu, Columbia, MD) with a Waters (Milford, MA) ACQUITY UPLC BEH C18 column (2.1 × 100 mm, 1.7 µm particle size) maintained at 55 °C and a 20 min aqueous acetonitrile gradient, at a flow rate of 250 µL/min. Solvent A was water with 0.1% formic acid and Solvent B was acetonitrile with 0.1% formic acid. The initial condition were 97% A and 3 % B, increasing to 45% B at 10 min, 75% B at 12 min where it was held at 75% B until 17.5 min before returning to the initial conditions. The eluate was delivered into an AB SCIEX TripleTOF™ 5600 System (QTOF) using a Duospray™ ion source (AB SCIEX, Framingham, MA). Purities of assayed compounds were in all cases greater than 95%, as determined by a Waters 2695 HPLC system (Waters, Milford, MA, U.S.A) with a Restek (Bellefonte, PA, U.S.A) HPLC C18 column (4.6 × 150 mm, 5 µm particle size) and a Viva C18 guard cartridge (10 × 4.0 mm, 5 µm particle size) with a 10 µL of injection volume. The mobile phase solvent A was 0.1% formic acid in water, and solvent B was 0.1% formic acid in acetonitrile. The gradient program was: 0-18 min, 5-45% B in A; 18-22 min, 45-90% B in A; 22-27 min, 90% B in A; 27-27.5 min, 90-5% B in A; 27.5-35 min, 5% B in A at a flow of 1 mL/min.

**NMR method section.** Each compound was dissolved in methanol-d4 or DMSO-d6. All NMR data were acquired at 298 K on Bruker Avance NEO 600 MHz spectrometer (Bruker Biospin, Rheinstetten, Germany) equipped with a 5 mm TCI cryoprobe. 1D $^{1}$H NMR spectra and a series of 2D NMR spectra, including $^{1}$H -$^{1}$H TOCSY, $^{1}$H -$^{1}$H COSY, $^{1}$H -$^{1}$H JRes, $^{1}$H -$^{13}$C HSQC, and $^{1}$H -$^{13}$C HMBC, were recorded and processed as previously described with some modifications.[56] The $^{1}$H and $^{13}$C chemical shifts were referenced to deuterated solvent residual proton signal at 3.31 ppm and $^{13}$C signal at 49.5 ppm in methanol-d4, respectively and at 2.50 ppm and 39.5 ppm in DMSO-d6, respectively. Coupling constants (J value) are reported in Hz. Spin multiplicities are described as s (singlet), br (broad singlet), d (doublet), t (triplet), and m (multiplet).

## Synthesis of Indolimines:

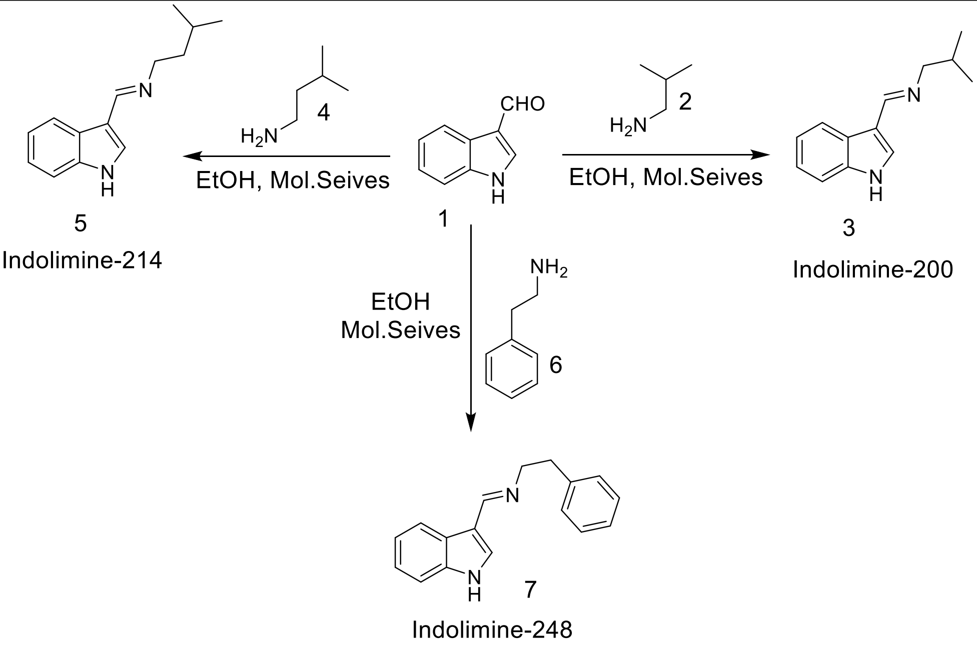


**Scheme 1. Synthesis of indolimines**

Indolimine-200, indolimine-214, and indolimine-248 were synthesized as shown in Scheme 1.

**Synthesis of (*E*)-1-(1H-indol-3-yl)-*N*-isobutylmethanimine (Indolimine-200) (3):**
Indol-3-carboxaldehyde **1** (500 mg, 3.44 mmol) and isobutylamine **2** (1.5 eq, 377 mg, 5.16 mmol) were dissolved in anhydrous ethanol (50 mL). Molecular sieves (5 g) were added, and the reaction mixture was refluxed for 7-8 hrs until the aldehyde completely disappeared (TLC). The reaction mixture was filtered through celite, and the excess isobutylamine was removed by rotavaporation, and the product was dried in a high vacuum pump to obtain (indolimine-200) **3** (660 mg, yield 95%) as a brown viscous oil. The identity of indolimine-200 was confirmed by nuclear magnetic resonance (NMR) and mass spectra analysis. $^{1}$H-NMR (600 MHz, MeOD, 25 ºC): δ 8.46 (s, 1H, N=CH), 8.08 (m, 1H, =CH), 7.70 (s, 1H, =CH-N), 7.41 (m, 1H, =CH), 7.19 (m, 1H, =CH), 7.14 (m, 1H, =CH), 3.40 (dd, *J* = 6.72 Hz, 1.16 Hz, 2H, CH2), 1.99 (m, 1H, CH), 0.99 (d, *J* = 6.72 Hz, 6H, CH3); $^{13}$C-NMR (150 MHz, MeOD, 25 ºC): δ 158.78, 139.2, 131.45, 127.07, 124.03, 121.97, 121.68, 115.5, 113.01, 70.7, 31.16, 21.1.

**Synthesis of (*E*)-1-(1H-indol-3-yl)-*N*-isopentylmethanimine (Indolimine-214) (5):**
Compound **5** was synthesized by following a similar experimental procedure as used above for compound **3,** starting from indol-3-carboxaldehyde **1** (500 mg, 3.44 mmol) and isopentylamine **4** (1.5 eq, 450 mg, 5.16 mmol). Indolimine-214 was characterized by NMR and mass spectra analysis (brown viscous oil, 690 mg, yield 93%). $^{1}$H-NMR (600 MHz, DMSO-d6, 25 ºC): δ 11.43

(br, 1H, NH), 8.45 (s, 1H, N=CH), 8.19 (m, 1H, =CH), 7.72 (s, 1H, =CH-N), 7.41 (m, 1H, =CH), 7.15 (m, 1H, =CH), 7.08 (m, 1H, =CH), 3.52 (dt, *J* = 7.15 Hz, 1.1 Hz, 2H, CH2), 1.71 (m, 2H, CH2), 1.51 (q, *J* = 7.05 Hz, 1H, CH), 0.93 (d, *J* = 6.66 Hz, 6H, CH3); $^{13}$C-NMR (150 MHz, DMSO-d6, 25 ºC): δ 154.95, 136.83, 130.26, 124.94, 122.06, 121.4, 119.93, 114.4, 111.36, 59.24, 40.04, 25.09, 22.26.

**Synthesis of (*E*)-1-(1H-indol-3-yl)-*N*-phenethylmethanimine (Indolimine-248) (7):**
The experimental procedure used for synthesizing compound **7** was also the same as **3**. Compound **7** was prepared from indol-3-carboxaldehyde **1** (500 mg, 3.44 mmol) and phenethylamine **6** (1.5 eq, 625 mg, 5.16 mmol) in ethanol. The excess phenethylamine was removed by repeatedly washing the product with hexane and dried using a high vacuum pump to afford indolimine-248 as a brown viscous oil, and it was characterized by NMR and mass spectra analysis (760 mg, yield 88%). $^{1}$H-NMR (600 MHz, MeOD, 25 ºC): δ 8.33 (s, 1H, N=CH), 8.05 (m, 1H, =CH), 7.64 (s, 1H, =CH-N), 7.4 (m, 1H, =CH), 7.248 (m, 2H, =CH), 7.247 (m, 2H, =CH), 7.19 (m, 1H, =CH), 7.157 (m, 1H, =CH), 7.13 (m, 1H, =CH), 3.80 (dt, *J* = 7.3 Hz, 1.1Hz, 2H, CH2), 2.99 (t, *J* = 7.3 Hz, 2H, CH2); $^{13}$C-NMR (150 MHz, MeOD, 25 ºC): δ 159.16, 141.36, 139.03, 131.39, 130.3, 129.47, 127.2, 126.96, 123.9, 121.9, 121.61, 115.44, 112.89, 64.15, 39.01.

A

| Compound name | Formula | Ionization mode | Exact mass | LOD (nM) |
|---|---|---|---|---|
| Indolimine-200 | C13H16N2 | Positive | 200.1313 | 1 |
| Indolimine-214 | C14H18N2 | Positive | 214.1469 | 1 |
| Indolimine-248 | C17H16N2 | Positive | 248.1313 | 1 |

B

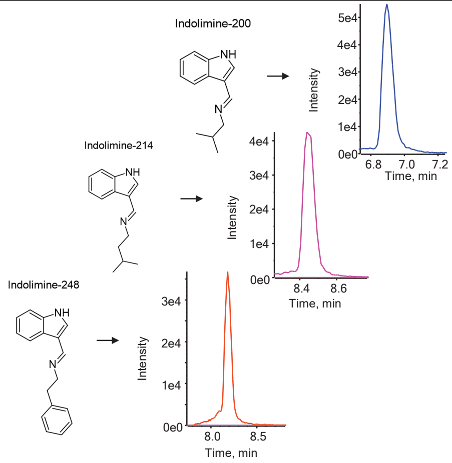


C

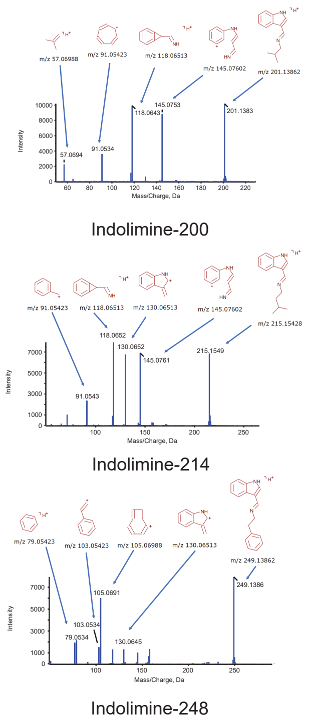


**Figure S1**. Identification of synthetic indolimines by LC-MS/MS. A, Overview of LC-MS/MS parameters of indolimines. B, Extracted ion chromatograms (EICs) of indolimines. C, Fragmentation annotations of the indolimines.

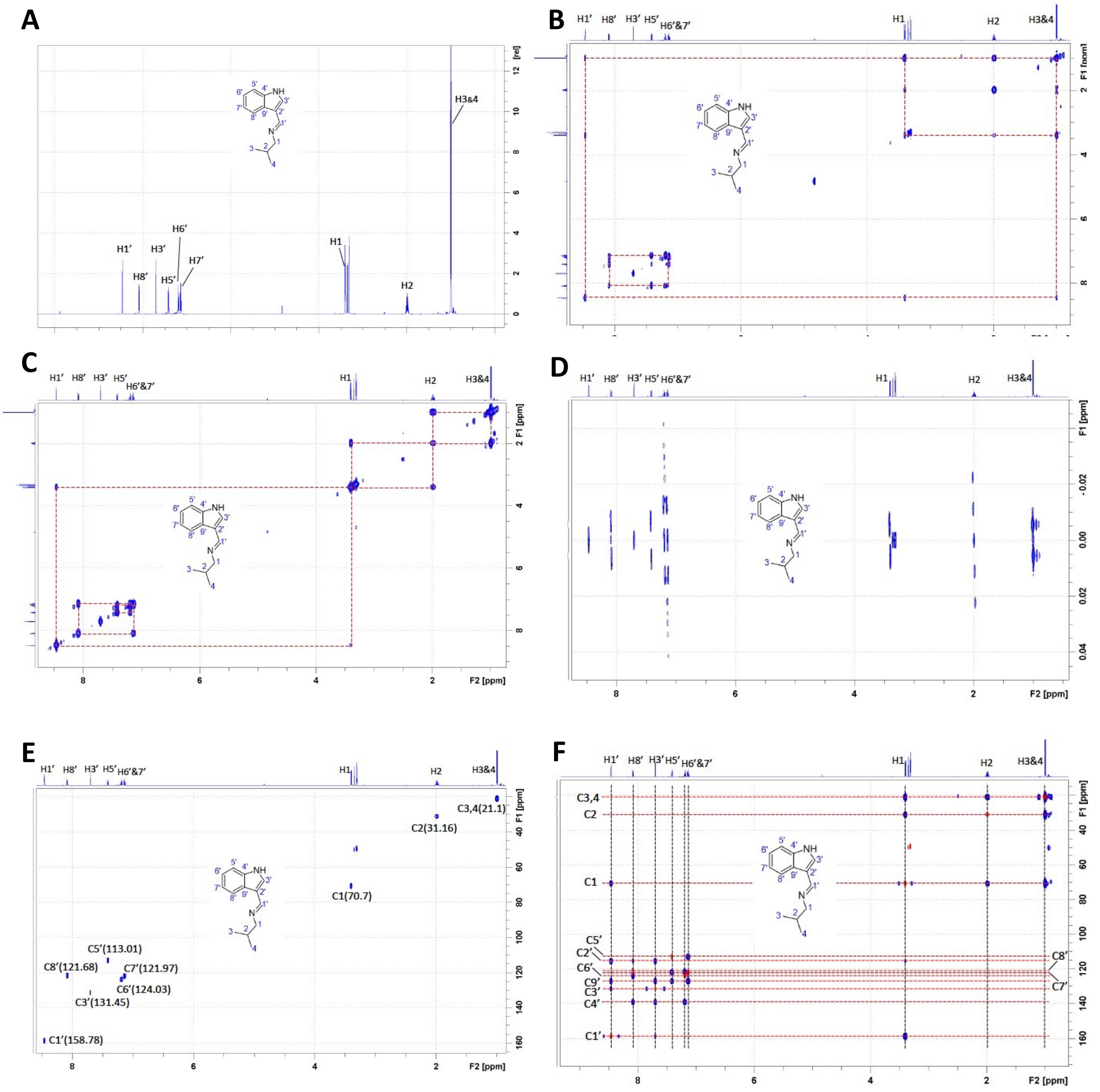


**Figure S2.** Conformation of the structure of indolimine-200 by NMR. (A) 1D $^{1}H$ spectrum of indolimine-200 with assignments. (B) $^{1}H$-$^{1}H$ TOCSY spectrum of indolimine-200 with signal assignments. (C) $^{1}H$-$^{1}H$ COSY spectrum of indolimine-200 with signal assignments. (D) $^{1}H$-$^{1}H$ JREs spectrum of indolimine-200 showing J-coupling patterns. (E) $^{1}H$-$^{13}C$ HSQC spectrum of indolimine-200 and signal assignments. (F) $^{1}H$-$^{13}C$ HMBC (blue) and HSQC (red) spectrum of indolimine-200 and signal assignments.

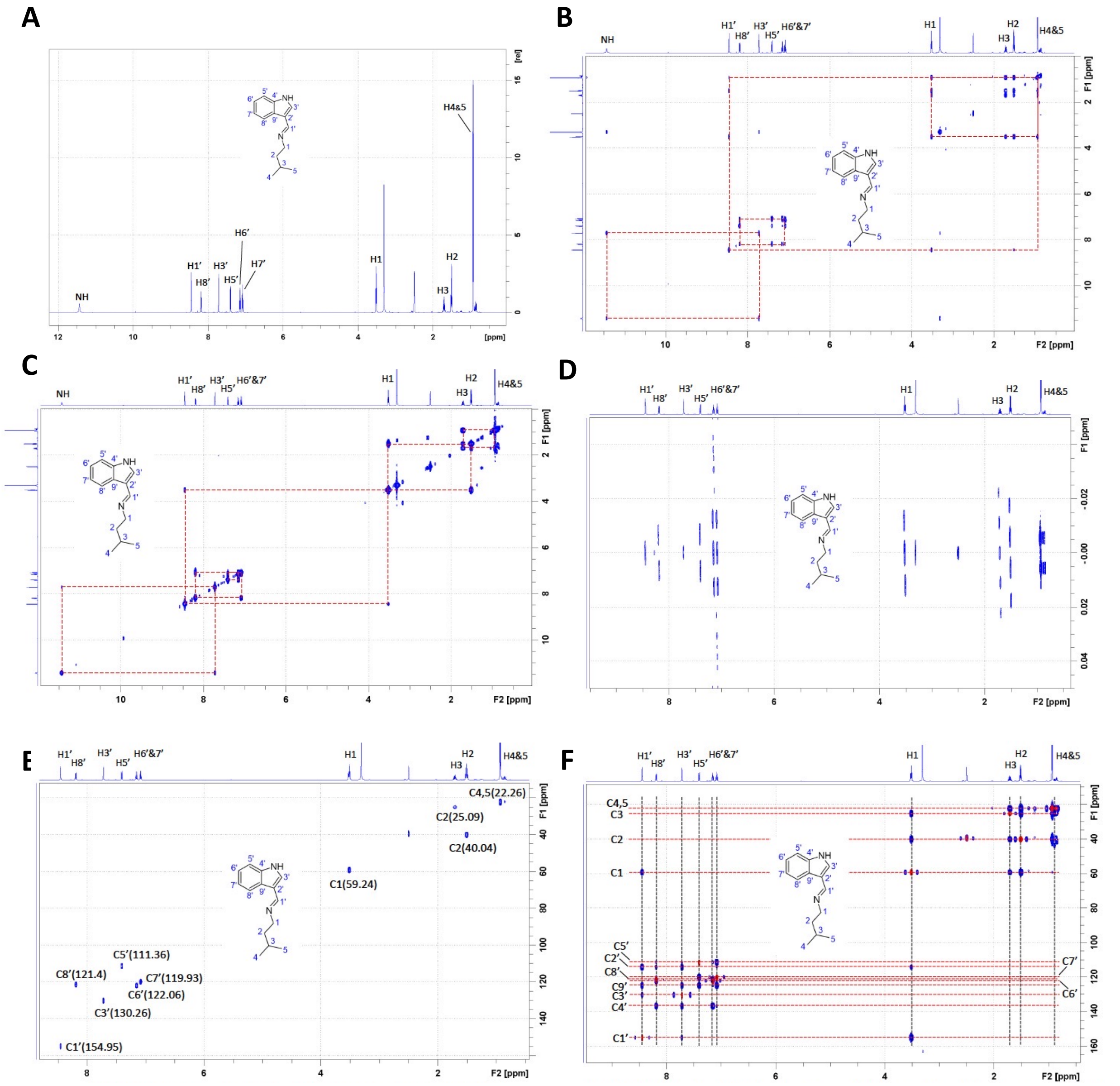

**Figure S3.** Conformation of the structure of indolimine-214 by NMR. (A) 1D $^{1}H$ spectrum of indolimine-214 with assignments. (B) $^{1}H$-$^{1}H$ TOCSY spectrum of indolimine-214 with signal assignments. (C) $^{1}H$-$^{1}H$ COSY spectrum of indolimine-214 with signal assignments. (D) $^{1}H$-$^{1}H$ JREs spectrum of indolimine-214 showing J-coupling patterns. (E) $^{1}H$-$^{13}C$ HSQC spectrum of indolimine-214 and signal assignments. (F) $^{1}H$-$^{13}C$ HMBC (blue) and HSQC (red) spectrum of indolimine-214 and signal assignments.

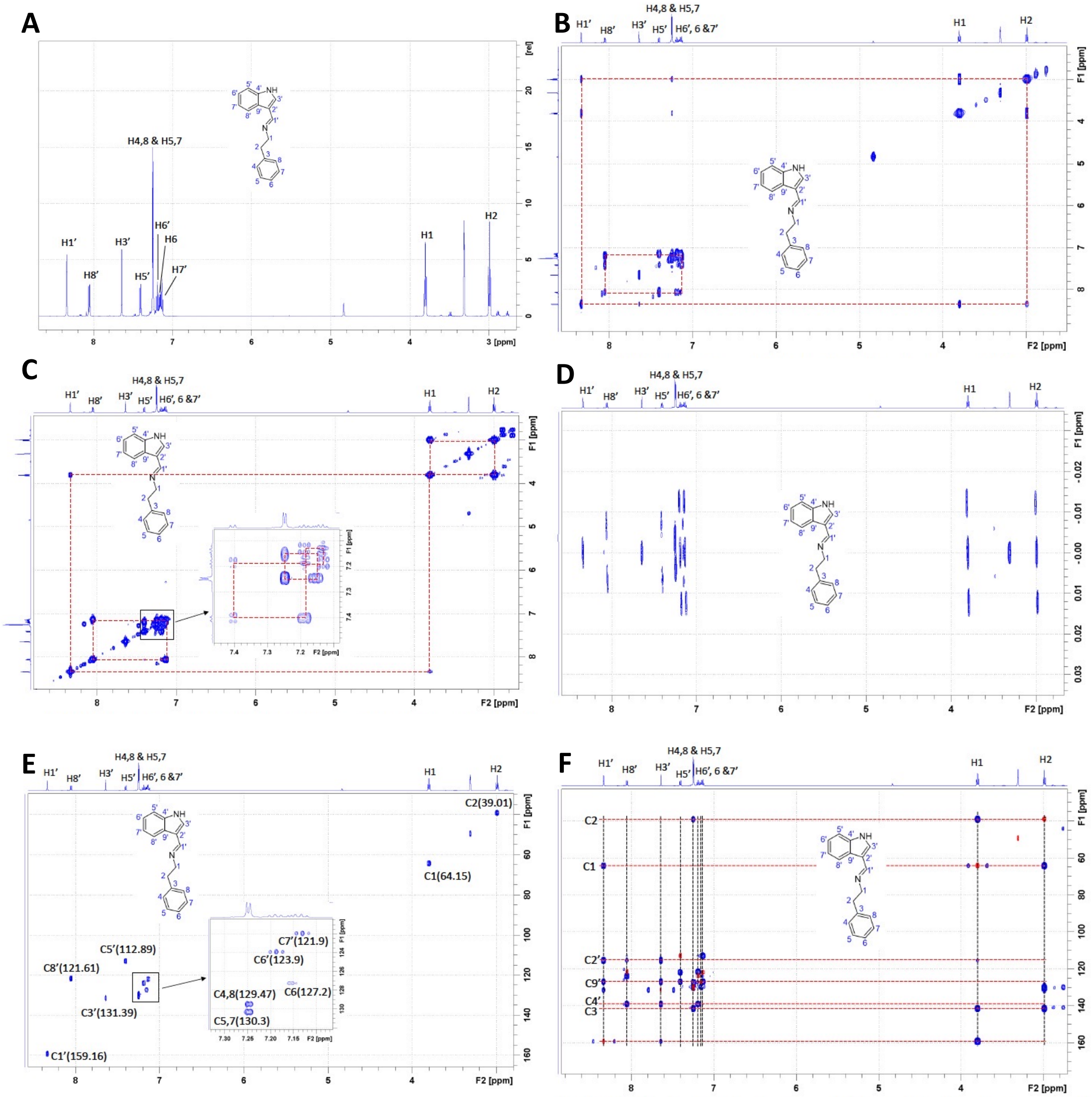


**Figure S4.** Conformation of the structure of indolimine-248 by NMR. (A) 1D $^{1}H$ spectrum of indolimine-248 with assignments. (B) $^{1}H$-$^{1}H$ TOCSY spectrum of indolimine-248 with signal assignments. (C) $^{1}H$-$^{1}H$ COSY spectrum of indolimine-248 with signal assignments. (D) $^{1}H$-$^{1}H$ JREs spectrum of indolimine-248 showing J-coupling patterns. (E) $^{1}H$-$^{13}C$ HSQC spectrum of indolimine-248 and signal assignments. (F) $^{1}H$-$^{13}C$ HMBC (blue) and HSQC (red) spectrum of indolimine-248 and signal assignments.

**Supplemental Table S1. Primer sequences utilized in qRT-PCR analysis.**

| Gene | Forward sequence (5´→3´) | Reverse sequence (5´→3´) |
|---|---|---|
| *ACTB* | *caccattggcaatgagcgattc* | *aggtctttacggatatccacat* |
| *CYP1A1* | *acctcagcagccacctccaagat* | *gaggtcttgaggccctgat* |
| *AHRR* | *gtgcgaatcggaactgcatggaaa* | *tcagtctgttccctgagcaccaaa* |
| *PARP7* | *gattctcaggagcacttggaaag* | *tggtgtggacagccttgctagt* |
| *Il6* | *agacagccactcaggtcttca* | *ttctgccagtgcctctttactg* |

**Supplemental Table S2. Computational Validation of the human AHR PAS-B domain model.**

| Model | Active Site Cavity Volume ($Å^3$) [a] | Test Ligand | ANF Binding Energy (kcal/mol) [b] | ANF Re-Docking Error RMSD (Å) [c] |
|---|---|---|---|---|
| **Human AHR** PAS B domain model PDB: 7ZUB | 830 | Indirubin | -12.8 | 0.498 |

[a] Active site volume or cavity size (in angstroms) as calculated using the program Caver Web 1.0.

[b] Autodock Vina Binding Energy for Indirubin in a 30 $Å^3$ grid centered on the PAS-B domain of the AHR (7SUB).

[c] RMSD deviation for Indirubin re-docking solutions in structure-based models in Autodock Vina, as calculated using LigRMSD. Models with a ligand re-docking RMSD error of less than 2.0 Å are generally considered acceptable for our docking protocol.
